# Visual short-term memory deficits in REM sleep behaviour disorder mirror those in Parkinson’s disease

**DOI:** 10.1093/brain/awv334

**Published:** 2015-11-18

**Authors:** Michal Rolinski, Nahid Zokaei, Fahd Baig, Kathrin Giehl, Timothy Quinnell, Zenobia Zaiwalla, Clare E. Mackay, Masud Husain, Michele T. M. Hu

**Affiliations:** ^1^ 1 Oxford Parkinson’s Disease Centre (OPDC), University of Oxford, Oxford, UK; ^2^ 2 Nuffield Department of Clinical Neurosciences, University of Oxford, Oxford, UK; ^3^ 3 Department of Psychiatry, University of Oxford, Oxford, UK; ^4^ 4 Department of Experimental Psychology, University of Oxford, Oxford, UK; ^5^ 5 Department of Nuclear Medicine, University of Cologne, Kerpener Straße 62, 50937 Cologne, Germany; ^6^ 6 Respiratory Support and Sleep Centre, Papworth Hospital, Cambridge, UK; ^7^ 7 Department of Clinical Neurophysiology, John Radcliffe Hospital, Oxford, UK

**Keywords:** Parkinson's disease, REM sleep behaviour disorder, biomarkers, memory, attention

## Abstract

Individuals with REM sleep behaviour disorder are at significantly higher risk of developing Parkinson’s disease. Here we examined visual short-term memory deficits—long associated with Parkinson’s disease—in patients with REM sleep behaviour disorder without Parkinson’s disease using a novel task that measures recall precision. Visual short-term memory for sequentially presented coloured bars of different orientation was assessed in 21 patients with polysomnography-proven idiopathic REM sleep behaviour disorder, 26 cases with early Parkinson’s disease and 26 healthy controls. Three tasks using the same stimuli controlled for attentional filtering ability, sensorimotor and temporal decay factors. Both patients with REM sleep behaviour disorder and Parkinson’s disease demonstrated a deficit in visual short-term memory, with recall precision significantly worse than in healthy controls with no deficit observed in any of the control tasks. Importantly, the pattern of memory deficit in both patient groups was specifically explained by an increase in random responses. These results demonstrate that it is possible to detect the signature of memory impairment associated with Parkinson’s disease in individuals with REM sleep behaviour disorder, a condition associated with a high risk of developing Parkinson’s disease. The pattern of visual short-term memory deficit potentially provides a cognitive marker of ‘prodromal’ Parkinson’s disease that might be useful in tracking disease progression and for disease-modifying intervention trials.

## Teaser

Individuals with REM sleep behaviour disorder (RBD) are at high risk of Parkinson’s disease. Rolinski, Zokaei *et al.* show that they also display the same pattern of visual short-term memory deficits as patients with Parkinson’s disease, and suggest that this ‘fingerprint’ of memory impairment could be a prodromal disease marker.

## Graphical abstract

**Figure:**
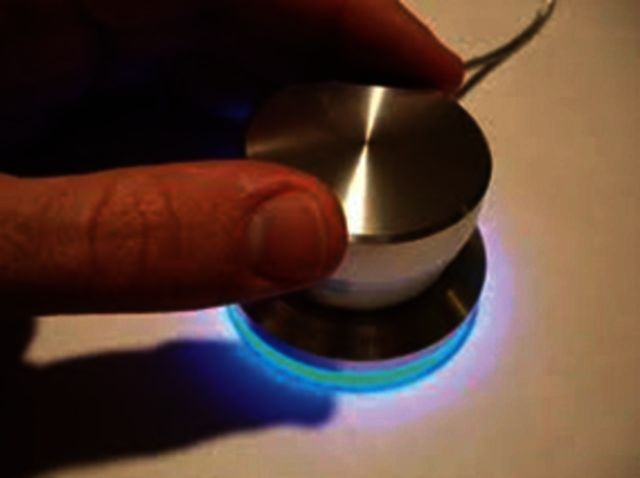
Individuals with REM sleep behaviour disorder (RBD) are at high risk of Parkinson’s disease. Rolinski, Zokaei *et al.* show that they also display the same pattern of visual short-term memory deficits as patients with Parkinson’s disease, and suggest that this ‘fingerprint’ of memory impairment could be a prodromal disease marker.

## Introduction

Prodromal Parkinson’s disease—the period between the onset of neurodegeneration and diagnosis—is likely to be the optimal time for introduction of potential curative or disease-modifying treatments. It is associated with several symptoms, including cognitive ones, which might provide a means for early detection of Parkinson’s disease. However, because screening for such deficits on a population-wide basis is challenging and unlikely to be a viable strategy, a better understanding of prodromal Parkinson’s disease might emerge from targeting ‘enriched’ at-risk cohorts instead ( Berg *et al.* , 2012 ).

One such group is individuals with idiopathic rapid eye movement (REM) sleep behaviour disorder, commonly referred to as RBD. RBD is a parasomnia characterized by motor behaviours associated with vivid dreams during REM sleep ( Boeve, 2010 ). Prospective cohort studies have observed a very strong association between idiopathic RBD and subsequent clinically defined neurodegenerative disease, with up to 80% of cases affected ( Postuma *et al.* , 2009 ; Boot *et al.* , 2012 ; Schenck *et al.* , 2013 ). While some patients develop dementia with Lewy bodies or multiple system atrophy, most eventually develop Parkinson’s disease ( Boeve, 2010 ). This high risk of conversion makes patients with RBD ideal candidates for neuroprotective trials against Parkinson’s disease ( Postuma *et al.* , 2015 ).

A few studies have reported cognitive deficits in RBD, including modest or no impairments on short-term or working memory tests that measure ‘span’ or number of items that individuals can retain ( Massicotte-Marquez *et al.* , 2008 ; Fantini *et al.* , 2011 ). However, traditional span measures rely on a binary response: either something is remembered correctly or it is not. But just because an individual fails to recall an item does not necessarily mean that it was completely lost from memory. Recently, an alternative theoretical and empirical approach has been developed to investigate the resolution or precision with which items are retained. Rather than simply asking whether an item is remembered or not (for a review see Ma *et al.* , 2014 ), this approach provides a more sensitive measure of visual short-term memory (VSTM) performance than span, including in patients with Parkinson’s disease ( Zokaei *et al.* , 2015 ).

Importantly, tasks that measure precision of recall also provide a means to dissect out sources of error contributing to the pattern of performance using modern statistical techniques ( Bays *et al.* , 2009 ). While it is known that many types of brain disorder can be associated with VSTM deficits, this might be due to different underlying mechanisms in different groups. In a recent study employing the same paradigm as used here, dissociable signature deficits associated with glucocerebrosidase ( *GBA* ) mutations—the highest known genetic risk factor for developing Parkinson’s disease—and sporadic Parkinson’s disease were reported ( Zokaei *et al.* , 2014 ). Specifically, while *GBA* -positive individuals showed increased misbinding errors (reflecting interference between items stored in memory), cases with sporadic Parkinson’s disease demonstrated increased random errors (guesses). *GBA* -positive cases with Parkinson’s disease showed both types of error. Crucially, there was no evidence that *GBA* -positive individuals without Parkinson’s disease have the same type of memory impairment as those with Parkinson’s disease. Here, we assessed VSTM in idiopathic, *GBA* -negative cases with RBD—who have a much higher risk of developing Parkinson’s disease than *GBA* -positive subjects—and asked whether any deficit in their memory mirrors the pattern observed in patients with established, sporadic *GBA* -negative Parkinson’s disease.

## Subjects and methods

### Participants

Twenty-one patients with RBD were recruited from sleep clinics at the John Radcliffe Hospital, Oxford and Papworth Hospital, Cambridge. The diagnosis of RBD was made on the basis of clinical and polysomnographic evidence, according to standard International Classification of Sleep Disorders-II criteria ( Lapierre and Montplaisir, 1992 ). RBD was defined as an increase in tonic or phasic chin EMG activity during REM sleep and either history of elaborate motor activity associated with dream content, or the characteristic behavioural manifestations occurring in REM sleep during polysomnographic recordings. Patients were excluded if their RBD was judged by their clinical team to be secondary to medication use or associated with other neurological conditions, including Parkinson’s disease and narcolepsy.

Fifteen non-medicated and 11 medicated patients with Parkinson’s disease, as well as 26 age-matched healthy individuals participated (see Table 1 for participants’ demographics). Ethical approval was given by the Oxford University Research Ethics Committee. Patients with Parkinson’s disease were recruited if they met the UK Parkinson’s Disease Society Brain Bank criteria for the diagnosis of idiopathic Parkinson’s disease ( Hughes *et al.* , 1992 ). All participants had normal or corrected-to-normal vision and normal colour vision. All patients with RBD and Parkinson’s disease were screened for common *GBA* mutations ( Supplementary material ) and control participants had no neurological disease or family history of Gaucher’s disease. All cases with Parkinson’s disease and RBD reported here were confirmed *GBA* -negative.

**Table 1:** Demographic information on all patient groups and healthy controls

	Healthy controls ( *n* = 26)	RBD patients ( *n* = 21)	PD patients ( *n* = 26)	Medicated PD ( *n* = 11)
			Non-medicated ( *n* = 15)	
Age	66 (7)	66 (9)	65 (7)	67 (6)
Gender (M/F)	18/8	19/2	9/6	6/5
Years of education	14.7 (3.3)	14.6 (3.5)	15 (3.4)	14 (3)
MMSE	29 (1.1)	27.8 (1.5)	28.9 (0.9)	28.0 (1.3)
Years of diagnosis	n/a	2.7 (1.9)	0.66 (0.74)	2.7 (1.3)
Daily levodopa equivalent dose	n/a	n/a	n/a	355 (152)
UPDRS III	n/a	n/a	13 (4)	15 (3)

Values are mean (SD). MMSE = Mini-Mental State Examination; n/a = not applicable; PD = Parkinson’s disease.

UPDRS = Unified Parkinson’s Disease Rating Scale.

### Visual short-term memory task

**Figure 1:**
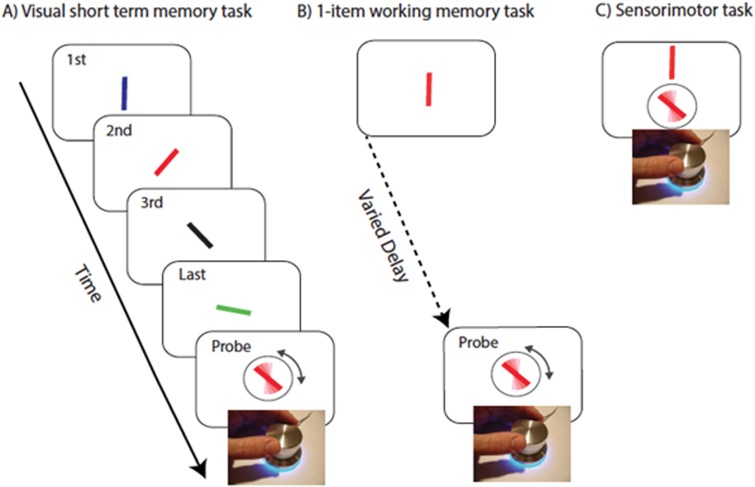
**Task to measure precision of recall.** ( **A** ) A sequence of four coloured oriented bars were presented sequentially. Any of the bars could be probed by colour of the response stimuli and participants were asked to adjust the orientation of the probed bar to the orientation of the bar with same colour (red in this example). ( **B** ) One-item working memory task. A rotating dial is used to orient the probe bar (surrounded by circle) to match the orientation of the probed bar presented following a delay. ( **C** ) Sensorimotor task. A rotating dial is used to orient the probe bar to match the orientation of the target bar presented above the probe and continuously on view.

The 4-item VSTM task was identical to that previously used by Zokaei *et al.* (2014) ( Fig. 1 A). Briefly, in each trial a sequence of four coloured bars of different orientation appeared on the screen centre and participants were asked to remember both the colour and orientation of the bars. At the end of each sequence, a randomly oriented probe bar of the same colour as one of the bars in the sequence was presented at screen centre. Participants were instructed to use a rotating dial to match the orientation of same coloured bar in the sequence. They clicked on the dial to confirm their selected orientation. Stimuli presented in any of the serial positions within the sequence were probed with equal probability and participants did not know beforehand which item would later be probed.

### Control tasks

Poor performance in the VSTM task might be attributed to factors other than the ability to maintain multiple items. To ensure various issues were not a concern for subsequent interpretation, three control tasks were administered: (i) pre-cueing: an identical design to the 4-item VSTM task but with 100% informative cues which tell the participant which colour will be probed; (ii) one-item VSTM with variable delays ( Fig. 1 B) to match durations between the probed item and appearance of the probe in VSTM task; (iii) sensorimotor task ( Fig. 1 C): participants simply match the orientation of a continuously presented bar using the response dial.

These tasks are identical to those previously used by Zokaei *et al.* (2014) and aim to control for deficits in attentional filtering, temporal decay of information and difficulties with dexterity in using the dial, respectively.

All tasks were presented on a laptop (32° × 19°) at ∼52 cm, in random order across participants. For each of the experimental VSTM, pre-cueing and 1-item VSTM tasks healthy controls completed 100–200 trials, patients with RBD completed 100 trials and patients with Parkinson’s disease completed 50–200 trials depending on their availability. All patients with Parkinson’s disease and RBD as well as 17 healthy controls completed 20 trials of the sensorimotor control task.

### Analysis

Recall precision was used as an overall measure of performance, calculated simply as the reciprocal of the circular standard deviation of response error (difference in response and target angle), with less variability corresponding to a more precise memory.

To identify mechanisms underlying VSTM impairments associated with RBD and Parkinson’s disease, we fit a probabilistic model that dissociates different sources of error in memory ( Bays *et al.* , 2009 ). In tasks similar to the one employed here, several sources of error can contribute to impaired performance ( Ma *et al.* , 2014 ). Error can arise due to (i) increased variability in memory for the orientation of the probed (target) item; (ii) increase in random responses; or (iii) systematic interference by other items retained in VSTM—these are responses centred on other, non-probed items in the memory array (‘misbinding’ errors; see Supplementary Fig. 1 for a schematic presentation of the model). Maximum posteriors for three sources of error were estimated using the MemToolbox ( Suchow *et al.* , 2013 ) (memtoolbox.org).

## Results

There was no significant difference in age and years of education between the three groups. However, both patients with RBD and Parkinson’s disease scored significantly worse than healthy controls on the Mini-Mental State Examination (MMSE) (Mann-Whitney U = 131, *P* = 0.002 and U = 232.5, *P* = 0.042, respectively), although on average both groups scored higher than 27 (a cut-off for mild cognitive impairment) on this measure. Furthermore, there was no significant correlation between MMSE and any of the measures of interest reported below. Specifically there was no significant correlation between MMSE and proportion of random responses either within each patient group or the combined group of participants.

### VSTM impairments in cases with RBD and Parkinson’s disease

**Figure 2:**
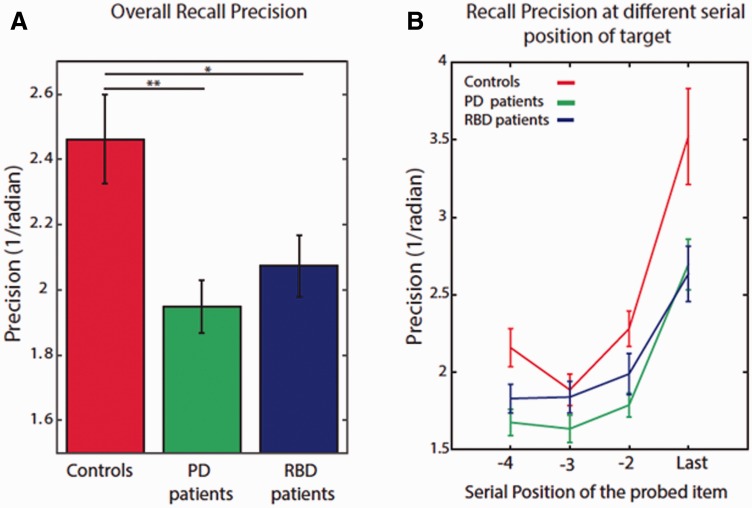
**Performance in the VSTM task.** ( **A** ) Overall recall precision in both patient groups was significantly worse compared to healthy controls. ( **B** ) This occurred at all serial positions of the probed item. PD = Parkinson’s disease.

There was no difference in overall VSTM performance between non-medicated and medicated patients with Parkinson’s disease and hence these two groups were collapsed for analysis ( *n* = 26). ANOVA with serial position of probe as within subject factor and group as between subject factor yielded a main effect of group [ *F* (2,70) = 6.3, *P* = 0.003]; compared to healthy participants, overall performance was significantly worse (i.e. less precise) in patients with RBD [ *t* (42.4) = 2.3, *P* = 0.025] as well as cases with Parkinson’s disease [ *t* (40.7) = 3.2 *P* = 0.003, Fig. 2 B]. Moreover, there was a significant effect of serial position on recall precision, showing the well-known effect of recency [degrees of freedom were corrected using Greenhouse-Geisser estimate; *F* (1.53,107) = 66, *P* < 0.001].

### Sources of error in VSTM

**Figure 3:**
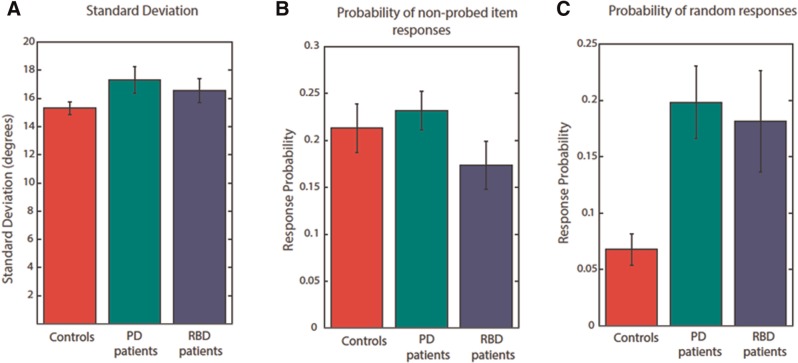
**Model estimates for different sources of error in VSTM performance.** ( **A** ) Concentration parameter (κ) did not differ significantly between patient groups and controls **.** ( **B** ) Probability of non-probed responses (misbinding errors) did not differ between groups. ( **C** ) Probability of random responses was significantly higher in both patients with Parkinson’s disease (PD) and RBD compared to controls.

Although both patient groups performed worse than healthy participants, the overall VSTM performance is not informative of the source of error—the pattern of deficit—in these disorders. To quantify the possible sources of error, we next applied a statistical mixture model of responses error. The results demonstrated no effect of group on variability in memory for the probed orientation ( Fig. 3 A) or on proportion of misbinding errors ( Fig. 3 B). However, there was a significant effect on proportion of random responses [Welch’s adjusted F ratio: *F* (2,36) = 8.8 *P* = 0.001]. Compared to healthy controls, both patients with RBD and Parkinson’s disease made significantly more random responses [ *t* (33.9) = 3.7, *P* = 0.001 and *t* (23.8), *P* = 0.024, respectively]. This was accompanied by a significant main effect of group on proportion of responses to the probed item [ *F* (2,72) = 5.2, *P* = 0.008]. There was no significant difference in all model estimates, between patients with Parkinson’s disease and RBD.

### Performance in control tasks

There were no significant differences in performance on the sensorimotor control task between the three groups ( Fig. 1 C). This is important in excluding impaired dexterity as a confounding factor in the 4-item VSTM task results, particularly for the Parkinson’s disease group. There were also no significant differences between the patient groups and healthy controls in the pre-cueing and 1-item ( Fig. 1 B) VSTM tasks. Any significant effect observed in the 4-item VSTM task therefore cannot be attributed simply to deficits in attending to items presented sequentially or in temporal decay of information. These findings also make it unlikely that excessive sleepiness in RBD is the reason for the deficit observed on the 4-item VSTM task.

## Discussion

The findings presented here demonstrate for the first time, to the best of our knowledge, that patients with RBD—at high risk for developing Parkinson’s disease—show deficits in VSTM identical to those observed in Parkinson’s disease. Specifically, deficits in recall precision in both patient groups are due to random corruption of memory, suggesting that they share the same underlying impairment in memory. The results of control experiments show that this is independent of sensorimotor deficits, difficulties in attending to different serial positions in a sequence or temporal decay of information, which makes it unlikely that excessive sleepiness in RBD is the reason for the deficit observed on the 4-item VSTM task.

Importantly, the paradigm used here allowed us to analyse the sources of error in performance ( Ma *et al.* , 2014 ). Recall error can firstly arise due to increase in variability in memory for the probed feature, that is, how well the probed feature is reproduced. Secondly, participants may make random responses, guessing the orientation of the probed bar, possibly due to failures at encoding or retrieval. Lastly, VSTM precision may be affected by misbinding errors. Unlike random responses, misbinding errors have been linked to hippocampal and medial temporal lobe pathology, including Alzheimer’s disease ( Parra *et al.* , 2009 ; Pertzov *et al.* , 2013 ) and mutations in GBA ( Zokaei *et al.* , 2014 ).

Individuals with Parkinson’s disease made significantly more random responses than controls, but importantly not significantly more misbinding errors, replicating and strengthening previous findings using this task ( Zokaei *et al.* , 2014 ). The same pattern of results was also present in patients with RBD. Although the precise mechanism underlying this type of error is yet to be established, it might be due to increased noise within neuronal networks involved in encoding or maintaining information. This could potentially arise due to cholinergic disruption in Parkinson’s disease ( Kehagia *et al.* , 2010 ; Hasselmo and Sarter, 2011 ) with associated fluctuations in attention leading to encoding or retrieval failure and therefore guessing on some trials ( Hasselmo and Sarter, 2011 ). Increased random responses could also be a consequence of dopaminergic dysfunction, associated with lower neural signal-to-noise ratio ( Sawaguchi and Goldman-Rakic, 1991 ; Kroener *et al.* , 2009 ). Indeed, improvements in VSTM performance on this task have now been reported in patients with Parkinson’s disease treated with dopaminergic drugs ( Zokaei *et al.* , 2015 ).

Dysfunction within both cholinergic and dopaminergic systems has now been reported in RBD before the onset of clinically defined neurodegenerative disease. Single-photon emission computed tomography (SPECT) has demonstrated decreased ^123^ I-FP-CIT uptake in the striatum of cases with RBD, with ∼40% of patients having an abnormal scan ( Selikhova *et al.* , 2009 ). Similarly, decreased ^11^ C-dihydrotetrabenazine ( ^11^ C-DTBZ) striatal binding on PET scanning points to loss of dopaminergic neurons in RBD ( Ferini-Strambi *et al.* , 2004 ). Although evidence for cholinergic dysfunction is scarcer, one PET study has revealed reduced acetylcholinesterase activity in RBD ( Valerio *et al.* , 2013 ).

A number of previous studies have reported cognitive impairment in RBD ( Ferini-Strambi *et al.* , 2004 ; Massicotte-Marquez *et al.* , 2008 ; Terzaghi *et al.* , 2008 ), which has been shown to be progressive ( Fantini *et al.* , 2011 ). Moreover, the presence of RBD in established Parkinson’s disease is associated with increased frequency of cognitive impairment ( Rolinski *et al.* , 2014 ), and greater risk of dementia ( Postuma *et al.* , 2012 ). These pioneering studies have assessed several cognitive domains in RBD, but the mechanisms underlying the observed impairments and their relationship to sporadic Parkinson’s disease remain poorly understood. The more focused approach used here implicates a similar mechanism underlying VSTM deficits in Parkinson’s disease and RBD. Importantly, as patients with RBD were not impaired in any of the control experiments, sleep disturbances in these patients is unlikely to explain the pattern of results. It is possible that increased random responses on the VSTM task might reflect a general cognitive decline in both patients with Parkinson’s disease and RBD. However, we did not find any significant correlation with MMSE scores.

The results presented here in RBD are in contrast to those found in individuals with *GBA* mutations, who carry the strongest genetic risk factor for developing Parkinson’s disease ( Neumann *et al.* , 2009 ; Sidransky *et al.* , 2009 ). On the paradigm used here, it has previously been shown that asymptomatic *GBA* -positive individuals are more likely to make misbinding errors than controls ( Zokaei *et al.* , 2014 ). Their pattern of deficit is different to that of cases with Parkinson’s disease who show increased random corruption of VSTM. Patients with Parkinson’s disease who are also *GBA* -positive appear to have a double hit, showing both increased misbinding and random corruption of VSTM ( Zokaei *et al.* , 2014 ). Although Parkinson’s disease is a heterogeneous condition and there might not be a ‘typical’ phenotype ( Selikhova *et al.* , 2009 ), our results suggest that RBD is more representative of prodromal stages of sporadic Parkinson’s disease than GBA because both RBD and Parkinson’s disease are associated with the same type of VSTM deficit. Hence RBD might be a better candidate disorder for clinical trials of novel disease-modifying interventions in Parkinson’s disease, potentially using the pattern of impairment identified here as a cognitive marker for incipient Parkinson’s disease in this group. Longitudinal studies are required to assess the feasibility of such an approach.

## Supplementary Material

Supplementary Data

## Abbreviations

RBD: REM sleep behaviour disorder
VSTM: visual short-term memory
