# Supplementary material for: Visual short-term memory deficits in REM sleep behaviour disorder mirror those in Parkinson’s disease
Source: Brain. 2015 Nov 18;139(1):47–53. doi: 10.1093/brain/awv334 (PMC4949392; doi:10.1093/brain/awv334)
Supplement: Supplementary Data [file awv334_supplementary_data.zip › brain-2015-01711-File002.pdf]

Supplementary material

Genetic testing

All patients, except for 7 PD patients that are discussed later, were screened for N370S and L444P mutations in the glucocerebrosidase (*GBA)* gene by extracting genomic DNA from blood samples using the AutoPure LS® (QIAGEN). PCR reactions were carried out with AmpliTaq Gold DNA polymerase (Applied Biosystems). Primer sequences used for N370S were: 5'-GCCTTTGTCCTTACCCTC*G -3' and 5'- GACAAAGTTACGCACCCAA-3'. A mismatch was engineered into the forward primer in order to create a XhoI restriction site in the PCR product from participants carrying the N370S mutation. For the L444P mutation primers used were: 5'-GGAGGACCCAATTGGGTGCGT-3' and 5'-ACGCTGTCTTCAGCCCACTTC-3'. The resulting PCR products were digested with XhoI (NEB) for N370S and NciI (NEB) for L444P and resolved by agarose gel electrophoresis. Mutations were then confirmed by sequencing. Briefly, DNA was treated with an ExoSAP reaction as follows: 1X SAP buffer, shrimp alkaline phosphatase (500 U; SAP, Promega), ExonucleaseI (2 U; NEB). Samples were incubated at 37°C for 1 hour and then 80 °C for 20 minutes. The sequencing reaction was performed according to BigDye Terminator v3.1 Cycle Sequencing protocol (Applied Biosystems). Following a clean up step, the sequencing read was performed on a 3700 DNA Analyser (Applied Biosystems) sequencing platform.

GBA mutation in 7 PD cases was screened by sequencing exons 1 to 11 of the GBA gene using previously published protocol (Zokaei et al., 2014). After amplification by PCR, the product was run on 1% agarose gel with ethidium bromide and size-checked to ensure intronic sequences using the Dye Terminator Sequencing Kit (Applied Biosystems) on an ABI 3700xl genetic analyser.

Analysis

Figure 1S is a schematic of the types of error associated with tasks similar to the one used here. Error can arise due to increased variability in memory for the orientation (Figure 1SA), increased misbinding errors (Figure 1SB) or increased random responses (Figure 1SC).

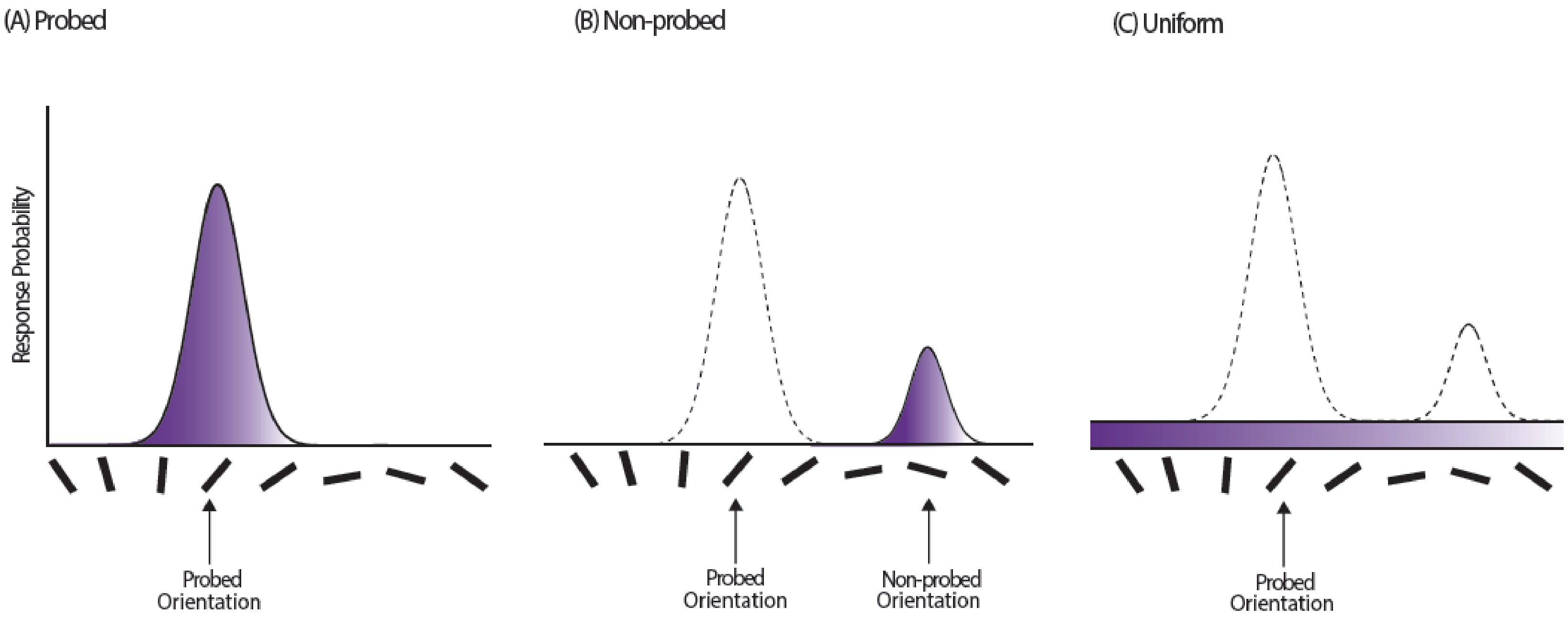

Figure 1S. Three sources of error in memory

Three possible sources of error that can corrupt in visual short-term memory. **A)** A Von Mises (circular Gaussian) distribution with concentration parameter κ, centred on the *probed* value, captures variability in memory for the probed orientation, with the area under the distribution (shaded) being proportional to the probability of responding to the *probe*. Effectively this is the noisiness of retaining the orientation of the probed item. **B**) Von Mises distribution with concentration parameter κ, centred on one of the *non-probed* items, i.e. one of the other bars shown in the memory array. The area under the distribution corresponds to the proportion of *non-probed* responses or *misbinding errors*. **C)** Uniform distribution of error corresponding to *random error*. Hence this distribution is constant across all possible orientations, regardless of whether they were actually presented in the memory array. The area under this distribution corresponds to the proportion of random responses.

References

Zokaei, N., McNeill, A., Proukakis, C., Beavan, M., Jarman, P., Korlipara, P., Hughes, D., Mehta, A., Hu, M.T.M., Schapira, A.H.V., et al. (2014). Visual short-term memory deficits associated with GBA mutation and Parkinson's disease. Brain J. Neurol. *137*, 2303–2311.
