# Supplementary figures and images for: Visual short-term memory deficits in REM sleep behaviour disorder mirror those in Parkinson’s disease
Source: Brain. 2015 Nov 18;139(1):47–53. doi: 10.1093/brain/awv334 (PMC4949392; doi:10.1093/brain/awv334)

## Slide 1
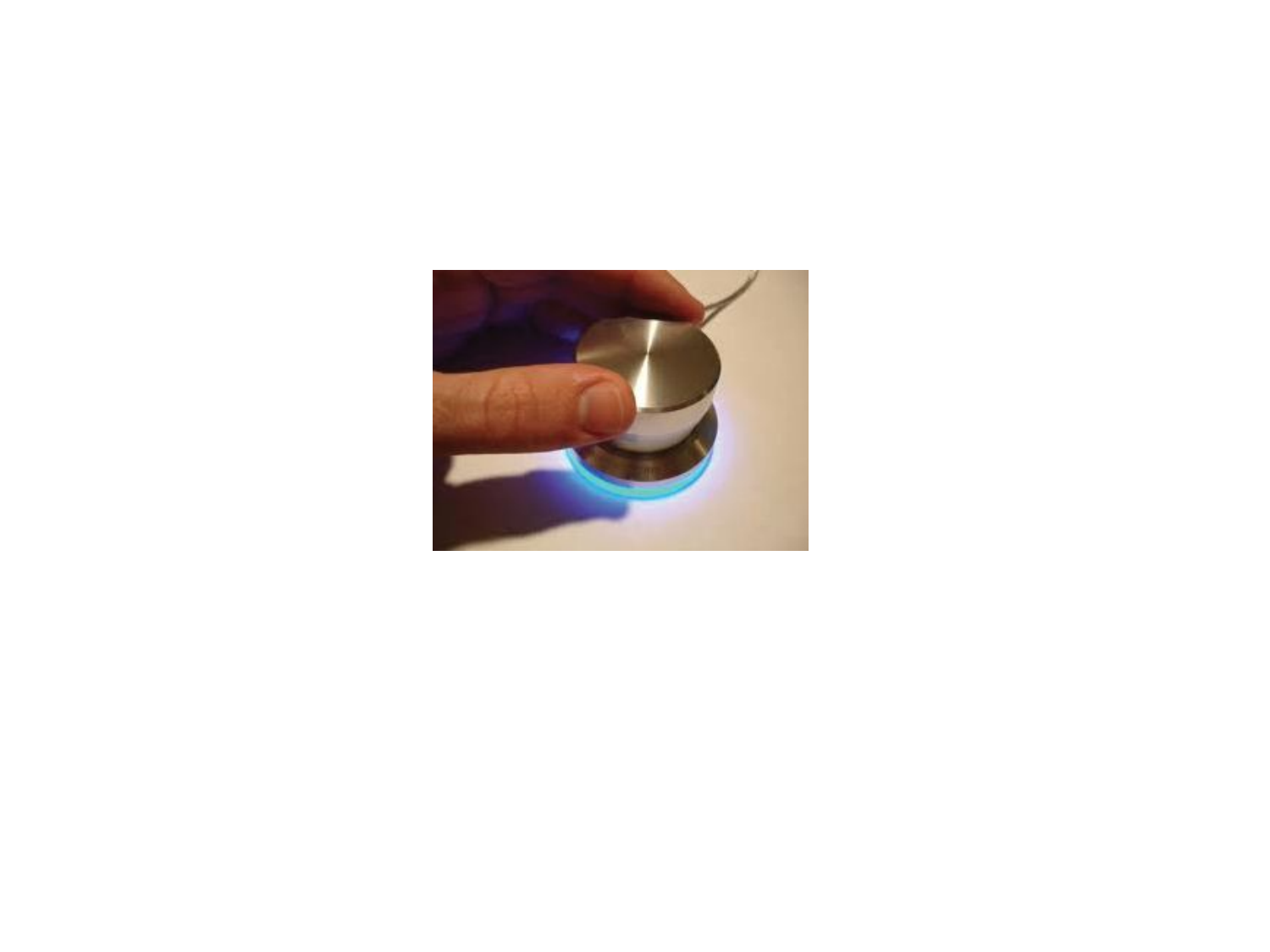

Supplement: Supplementary Data [file awv334_supplementary_data.zip › brain-2015-01711-File004.pptx]
